# Evaluation of patients' satisfaction after laparoscopic surgery in a tertiary hospital in Cameroon (Africa)

**DOI:** 10.11604/pamj.2017.28.216.11441

**Published:** 2017-11-08

**Authors:** Jovanny Tsuala Fouogue, Robert Tchounzou, Florent Ymele Fouelifack, Jeanne Hortence Fouedjio, Julius Sama Dohbit, Zacharie Sando, Emile Telesphore Mboudou

**Affiliations:** 1 Douala Gynaeco-Obstetric and Pediatric Hospital, Douala, Cameroon; 2 Higher Institute of Medical Technology, Yaoundé, Cameroon; 3 Faculty of Medicine and Biomedical Sciences of the University of Yaoundé I, Yaoundé, Cameroon

**Keywords:** Laparoscopy, surgery, patients, satisfaction, gynaecology

## Abstract

**Introduction:**

Access to laparoscopy is low in Cameroon where customers' satisfaction has not been reported so far. We assessed patients' satisfaction with the process of care during laparoscopic surgery in a new tertiary hospital.

**Methods:**

A questionnaire was addressed to consenting patients (guardians for patients under 18) with complete medical records who underwent laparoscopy at the Douala Gynaeco-Obstetric and Paediatric Hospital (Cameroon) from November 1, 2015 to July 31, 2016. The following modified Likert's scale was used to assess satisfaction: very weak: 0-2.5; weak 2.6-5; good: 5.1-7.5; very good: 7.6-10. Only descriptive statistics were used.

**Results:**

Response rate was 90% (45/50). Of the 45 respondents, 39 (86.7%) were female, 14(31.1%) were referred and 39 (86.7%) paid by direct cash deposit. Mean age was 36.8±11.9 years. Laparoscopies were carried out in emergency for 3 (6.7%) patients. Digestive abnormalities indicated 13 (28.9%) laparoscopies while gynaecologic diseases accounted for 32 (71.1%) cases. Perception of the overall care process was good with a mean satisfaction score of 6.8 ± 1.4. Scores in categories were: 0% (Very weak); 13.3% (weak); 57.8% (good) and 28.9% (very good). Specifically mean satisfaction scores were: 7.8 ± 1.0 with doctors' care; 7.1 ± 1.3 with hospital administration; 7.0 ± 1.2 with nursing and 4.7 ± 1.4 with the costs. Main complaints were: long waiting time (73.3%), constraining geographical access (66.7%) and expensiveness (48.9%).

**Conclusion:**

Patients were globally satisfied with the process of care but financial and geographical barriers should be addressed.

## Introduction

Over the past two decades, patients' satisfaction (PS) has emerged as an important indicator of health care quality [1,2]. PS can relate to the outcome of care (treatment) and/or to the perception of the process of care [3]. This applies to surgical care but is particularly true for laparoscopic surgery which has been proven to be superior to traditional open surgery in terms of minimal perioperative morbidity, lower overall costs, better cosmesis, reduced blood lost, reduced postoperative pain and hospital stay duration, with consequent quick postoperative recovery [4,5]. Laparoscopy has been introduced in Cameroon in 1992 and is currently practiced in five public and eight private hospitals [6-11]. To the best of our knowledge, no study has evaluated the satisfaction of patients with the process of care during laparoscopic surgery. In general, few studies have evaluated patients' satisfaction with health care in Cameroon, while it is a routine activity in developed countries [12,13]. In view of improving the process of care for patients undergoing laparoscopic surgery in Cameroon, we assessed their satisfaction in a tertiary hospital one year after its opening.

## Methods

**Study design and site:** We carried out a cross-sectional study at the Douala Gynaeco-Obstetric and Paediatric Hospital (DGOPH). This hospital was opened in September 2015. It is the largest health facility dedicated to mother and child's care in Cameroon (central Africa).

**Sampling and procedure:** Our sample was convenient. We consecutively included all consenting patients (legal guardians for those under 18) who underwent laparoscopic surgery from November 1, 2015 to July 31, 2016. Patients with incomplete medical records were excluded. Data were collected through a self-administered questionnaire on the following items: doctors' care, nursing, administrative procedures and cost. Participants were also asked to list their main complaints. Following a modified Likert's scale, participants were asked to score their satisfaction from 0 to 10 ("very weak": 0-2.5; "weak" 2.6-5; "good": 5.1-7.5; "very good": 7.6-10). Then a technical form was used to retrieve the followings parameters: age, sex, gravidity and parity (for women), marital status, mode of payment, occupational status, referral status, previous abdominopelvic surgery (laparoscopy or laparotomy), pregnancy status during laparoscopic surgery and its indication. Data were managed with Microsoft Office Excel^®^ (version 2010) software and descriptive statistics were computed.

**Ethics:** Our study was conducted in accordance with the Declaration of Helsinki on biomedical research involving human subjects. A clearance was obtained from the institutional committee of ethics of the DGOPH. (N°001/AR/HGOPED/DG/DM/SRFESS/pt). Written informed consent was obtained prior to participation from adult participants and from either parents or legal guardians of non-adult participants. Confidentiality was observed.

## Results

During the study period, 56 patients underwent laparoscopic surgery. For 6 of them, medical records were incomplete and 5 did not consent to participate (90% response rate).

**Baseline characteristics of patients and indications of laparoscopic surgeries:** Table 1 shows patients' characteristics. Mean age was 36.8 ± 11.9 years. One of the 39 (2.6 %) women had intra-uterine pregnancy (19 weeks and 3 days) during laparoscopy (indicated for ruptured hemorrhagic ovarian cyst). Twelve of our patients (26.7%) had had previous laparotomy and 5 (11.1%) had had previous laparoscopy. Referred patients made up 31.1 % (14 out of 45) of our sample. The financing was done by cash payment for 39 (86.7%) patients and by health insurance companies for 6 (13.3%) patients. Three (6.7%) laparoscopies were emergency procedures. Indications of laparoscopic surgeries are listed in Table 2.

**Table 1:** Baseline characteristics of patients (N = 45)

Characteristics	Frequencies	Proportions (%)
**Age (years)**		
≤ 15	1	2.2
16-30	11	24.4
31-45	25	55.6
≥ 46	8	17.8
**Sex**		
Male	6	13.3
Female	39	86.7
**Gravidity+**		
0-1	17	43.6
2-4	19	48.7
≥ 5	3	7.7
**Parity+**		
0	21	53.8
1-5	18	46.2
**Marital status**		
Married	31	68.9
Single	13	28.9
Widow (er)	1	2.2
**Occupational status**		
Student	3	6.7
Unemployed	9	20.0
Employed	32	71.1
Retired	1	2.2

+ Female patients only

**Table 2:** Indications of laparoscopic surgeries (N = 45)

Indications	Frequencies	Proportions (%)
**Digestive**	**13**	**28.9**
Chronic cholecystitis	4	8.9
GIT tumour	3	6.7
Ascites of unknown origin	1	2.2
Hiatal Hernia	1	2.2
Pancreatic tumour	1	2.2
Pathologic gastroesophageal reflux	1	2.2
Peptic pyloric stenosis	1	2.2
Symptomatic biliary sludge	1	2.2
**Gyneco-Obstetrical**	**32**	**71.1**
Infertility	19	42.2
Secondary infertility	14	31.1
Primary infertility	5	11.1
Post-myomectomy	5	11.1
Non ruptured ectopic pregnancy	2	4.4
Organic ovarian cyst	2	4.4
Chronic pelvic pain	2	4.4
CIN 3	1	2.2
Ruptured hemorrhagic ovarian cyst	1	2.2

CIN: Cervical Intra-epithelial Neoplasia; GIT: Gastro-Intestinal Tract

**Figure 1:**
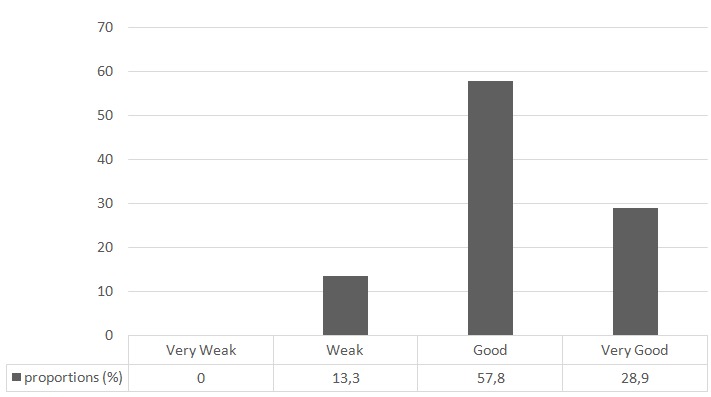
Overall satisfaction of patients after laparoscopic surgery (N=45)

**Patients' satisfaction and complaints after laparoscopic surgery:** Figure 1 shows the overall satisfaction of patients on a scale of 0 - 10. Mean satisfaction score was 6.8 ± 1.4. Table 3 illustrates the specific levels of patients' satisfaction and Table 4 lists their main complaints.

**Table 3:** Specific satisfaction of patients after laparoscopic surgery (0-10)

Items	Mean (SD)	Minimum	Maximum
Satisfaction with doctors’ care	7.8 (1.0)	5.5	10
Satisfaction with hospital administration	7.1 (1.3)	3.5	9
Satisfaction with nursing	7.0 (1.2)	4	9
Satisfaction with cost	4.7 (1.4)	2.5	8.5

SD: Standard deviation

**Table 4:** Main complaints of patients who underwent laparoscopic surgery (N = 45)

Patients’ complaints	N	%
Long waiting time	33	73.3
Constraining geographical accessibility	30	66.7
Expensiveness of services	22	48.9
Low-quality of meals	19	42.2
Insufficient communication	6	13.3
Red tape	5	11.1

## Discussion

Women predominated in our study (39/45; 86.7%). This is due to the fact that the study took place in a hospital dedicated to women and child health. The majority of participants was employed (32/45; 71.1%); this is understandable given that the study took place in the economic capital of the country. Infertility was the most frequent indication of laparoscopy (19/45; 42.2%). Similar findings are commonly reported in Cameroon for at least two reasons: laparoscopy was introduced in Cameroon three decades ago by gynaecologists; laparoscopic procedures indicated for infertility belong to the second levelof the European Society for Gynaecological Endoscopy classification for which local laparoscopic surgeons and gynaecologists are generally proficient [7, 14]. The level of overall satisfaction was either good or very good for 39 (86.7%) patients. This proportion seems high for a hospital in Cameroon where public health facilities are frequently described as "'patient-unfriendly"' [15]. The newness of the hospital in which the study was carried out, its referral status and the relatively small sample size may provide an explanation. More specifically, the average level of satisfaction with the costs was low (4.7 ± 1.4) and 22 (48.9%) participants complained about expensiveness. This is certainly due the very poor coverage (1%) by health insurance in Cameroon where most patients have to pay by direct cash deposit like in other in low-income African countries [15, 16]. It has been proven that in those countries, access to health services is inadequate because of financial barriers [17]. Indeed, over the past three decades, direct cash payment by users has partially replaced state funding of health care in Cameroon and sub-Saharan Africa [16]. Implementing low-cost laparoscopic surgery programme may reduce the costs of surgery [17]. Mean satisfaction scores with doctor's care, nursing and hospital administration were all above 7/10. This is contrary to the wide-spread poor perception of the quality of health care by users in sub-Saharan Africa [18]. Nevertheless, 33 (73.3%) complained of long waiting time. This was partly due to the fact that only one surgical team was available for each specialty (gynaecological and digestive). Even in developed countries the waiting time for elective laparoscopies is long [19]. Constraining geographical accessibility to the hospital was pointed out by 66.7% (30/45) of participants. Our study took place in Douala, the economic capital of Cameroon; like other sub-Saharan African capitals, the inadequacy of the road system results in permanent traffic jam that may have contributed to poor accessibility [20]. Despite the smallness of the sample size, our study provides a first insight of the patients' perception of the quality care in public hospitals in the sensitive domain of surgery. Moreover considering the current expansion of laparoscopic surgery in the country, it is crucial that all the stakeholders focus on quality care by investigating patients' perceptions and meeting their needs. Patients' satisfaction should be assessed on larger samples and outcomes of laparoscopic surgeries should also be studied in Cameroon.

## Conclusion

Patients were satisfied with the process of care during hospital stay for laparoscopic surgery. The costs, long waiting time and constraining geographic access were the main complaints.

### What is known about this topic

- Patients' satisfaction with laparoscopic surgery is good in developed countries where it is regularly assessed to maintain good quality of care;
- Satisfaction of Cameroonians patients with surgery in general and with laparoscopy in particular has not been assessed (to the best of our knowledge).

### What this study adds

- Our study gives a first appraisal of patients' satisfaction with laparoscopic surgery (they are globally satisfied);
- Expensiveness and poor geographical accessed are the main grievances reported by patients after laparoscopy.

## Competing interests

The authors declare no competing interests.
